# Genetic prion diseases presenting as frontotemporal dementia: clinical features and diagnostic challenge

**DOI:** 10.1186/s13195-022-01033-4

**Published:** 2022-06-29

**Authors:** Zhongyun Chen, Min Chu, Li Liu, Jing Zhang, Yu Kong, Kexin Xie, Yue Cui, Hong Ye, Junjie Li, Lin Wang, Liyong Wu

**Affiliations:** Department of Neurology, Xuanwu Hospital, Capital Medical University, Beijing, 100053 China

**Keywords:** Prion, Prion protein gene, Frontotemporal dementia, Creutzfeldt-Jakob disease

## Abstract

**Background:**

To elucidate the clinical and ancillary features of genetic prion diseases (gPrDs) presenting with frontotemporal dementia (FTD) to aid early identification.

**Methods:**

Global data of gPrDs presenting with FTD caused by prion protein gene mutations were collected from literature review and our records. Fifty-one cases of typical FTD and 136 cases of prion diseases admitted to our institution were included as controls. Clinical and ancillary data of the different groups were compared.

**Results:**

Forty-nine cases of gPrDs presenting with FTD were identified. Compared to FTD or prion diseases, gPrDs presenting with FTD were characterized by earlier onset age (median 45 vs. 61/60 years, *P* < 0.001, *P* < 0.001) and higher incidence of positive family history (81.6% vs. 27.5/13.2%, *P* < 0.001, *P* < 0.001). Furthermore, GPrDs presenting with FTD exhibited shorter duration (median 5 vs. 8 years) and a higher rate of parkinsonism (63.7% vs. 9.8%, *P* < 0.001), pyramidal signs (39.1% vs. 7.8%, *P* = 0.001), mutism (35.9% vs. 0%, *P* < 0.001), seizures (25.8% vs. 0%, *P* < 0.001), myoclonus (22.5% vs. 0%, *P* < 0.001), and hyperintensity on MRI (25.0% vs. 0, *P* < 0.001) compared to FTD. Compared to prion diseases, gPrDs presenting with FTD had a longer duration of symptoms (median 5 vs. 1.1 years, *P* < 0.001), higher rates of frontotemporal atrophy (89.7% vs. 3.3%, *P* < 0.001), lower rates of periodic short-wave complexes on EEG (0% vs. 30.3%, *P* = 0.001), and hyperintensity on MRI (25.0% vs. 83.0%, *P* < 0.001). The frequency of codon 129 Val allele in gPrDs presenting with FTD was significantly higher than that reported in the literature for gPrDs in the Caucasian and East Asian populations (33.3% vs. 19.2%/8.0%, *P* = 0.005, *P* < 0.001).

**Conclusions:**

GPrDs presenting with FTD are characterized by early-onset, high incidence of positive family history, high frequency of the Val allele at codon 129, overlapping symptoms with prion disease and FTD, and ancillary features closer to FTD. *PRNP* mutations may be a rare cause in the FTD spectrum, and *PRNP* genotyping should be considered in patients with these features.

**Supplementary Information:**

The online version contains supplementary material available at 10.1186/s13195-022-01033-4.

## Background

Frontotemporal dementia (FTD) is a group of early-onset dementia syndromes associated with underlying frontotemporal lobar degenerative pathology, which manifests as personality and behavioral changes, and impaired social cognition or language. It is a highly heritable group of neurodegenerative disorders, with roughly 30% of the patients having a strong family history [1–3]. The majority of the inherited cases of FTD involve autosomal dominant mutations in the chromosome 9 open reading frame 72 (*C9orf72*), progranulin (*GRN*), and microtubule-associated protein tau (*MAPT*) genes [4]. However, there is still a subset of FTD patients who may have genetic mutations linked to other neurodegenerative disorders [5, 6].

In recent years, an increasing number of cases clinically diagnosed as behavioral variant frontotemporal dementia (bvFTD) or primary progressive aphasia (PPA), the two subtypes of FTD, were identified as genetic prion diseases (gPrDs) caused by prion protein gene (*PRNP*) mutations following genetic testing. The *PRNP* P39L point mutation is strongly associated with the FTD phenotype and is therefore often misdiagnosed as FTD [7–9]. In addition, other point mutations or insertions/deletions of additional octapeptide repeat sequences in *PRNP* have been associated with the FTD phenotype [10–15]. The early presentation of frontotemporal symptoms and typical imaging changes related to FTD in *PRNP* mutation carriers, as well as the lack of typical symptoms or ancillary findings related to prion diseases, increases the risk of misdiagnosis. The exact clinical features of gPrDs presenting with FTD, and the features distinguishing them from classical FTD or prion diseases, remain unclear. It is therefore essential to identify patients with *PRNP* mutations that can be misdiagnosed as FTD. In addition, *PRNP* genotyping should be considered for patients with unexplained FTD syndrome.

Codon 129 is a determinant of the susceptibility and phenotype of prion diseases, as well as a possible risk factor for AD [16, 17]. In addition, it may alter the age of onset of FTD in some Caucasians [18], although it is unclear whether this codon plays a role in gPrDs presenting with FTD.

To address these issues, we conducted a systematic review to clarify the clinical features of gPrDs presenting as FTD. The patients with typical FTD and prion diseases were also compared to identify the similarities, overlaps, and differences between the two disorders, in order to improve early identification and reduce the risk of misdiagnosis.

## Methods

### Study design

A patient with a clinical diagnosis of bvFTD confirmed to be gPrDs (*PRNP* V180I) was enrolled at the Department of Neurology of Xuanwu Hospital. The PubMed, Embase, and Web of Science databases were searched in August 2021 for primary research articles and case studies reporting individuals carrying *PRNP* mutations and presenting with FTD features using the following keywords: (“frontotemporal lobar degeneration” OR “frontotemporal dementia” OR “primary progressive aphasia” OR “progressive non-fluent aphasia” OR “semantic dementia”) AND (“*PRNP*” OR “prion protein gene”). The titles and abstracts of each article were scanned independently by two authors (ZYC and MC) to exclude irrelevant studies. The full texts of the remaining studies were then retrieved, and those reporting individuals that were (1) positive for *PRNP* and (2) symptomatic with FTD features were selected. Publications not reporting original clinical data, including reviews, duplicate articles, and studies with patients lacking or unreported *PNRP* mutations, or inaccessible individual patient data were excluded. Any disagreement was resolved by discussion with a third author (JZ). Case series were excluded if patient characteristics were not individually accessible. A total of 665 relevant articles were identified in the initial search, of which 466 were retained after removing duplicate studies. Another 394 articles were eliminated based on titles and abstracts. After a thorough analysis of the remaining 72 full-text articles, 46 were ruled out for not meeting the inclusion criteria. Finally, 26 articles reporting 48 patients were included in the study. The flow chart of the search and selection procedure is shown in Additional file 1: Fig. S1.

Patients with definitive or probable FTD and patients with definitive or probable prion diseases that were admitted to the Department of Neurology at Xuanwu Hospital were consecutively recruited between July 1, 2014, and January 31, 2021. The diagnosis of probable bvFTD was made on the basis of consensus criteria published in 2011, which entails 3 of 6 clinical discriminatory features (disinhibition, apathy, loss of empathy, stereotyped/perseverative behavior, alterations in food preferences, and executive deficits), functional impairment, and neuroimaging features [19]. The diagnosis of PPA was made according to the criteria for PPA published in 2011 [20]. The cases with a known pathogenic mutation were classified as definite bvFTD or PPA. Patients with FTD-amyotrophic lateral sclerosis (ALS) met El Escorial’s criteria for ALS in addition to meeting the diagnostic criteria for bvFTD [21]. Creutzfeldt-Jakob disease (CJD) was diagnosed according to the updated clinical diagnostic criteria for CJD published in 2009 [22] and validated by the current WHO criteria [23]. Fatal familial insomnia (FFI) was diagnosed according to an Expert Consensus on Clinical Diagnostic Criteria for FFI [24]. Gerstmann-Straussler-Scheinker disease (GSS) was diagnosed if the patient had neuropsychiatric symptoms and a known *PRNP* mutation locus associated with GSS. Patients who lacked comprehensive medical records were excluded.

### Clinical and laboratory data

The following demographic and clinical variables were extracted from the retrieved articles: age at onset (years), gender, duration of symptoms (years), family history (the patient’s blood relatives have experienced comparable symptoms or have been diagnosed with prion diseases/FTD), initial symptoms, neurological manifestations during the clinical course (inhibition, apathy/inertia, loss of empathy/compassion, perseverative/compulsive behavior, hyperactivity, executive difficulties, cognitive dysfunction, cerebellar signs, pyramidal signs, extrapyramidal signs, myoclonus and visual signs), and auxiliary examination results (periodic sharp wave complexes (PSWCs) on electroencephalogram (EEG), cerebrospinal fluid (CSF) 14-3-3 and tau protein, structural neuroimaging and radionuclide neuroimaging, and neuropathological findings). Patients were further grouped on the basis of codon 129 genotypes (Met/Met (MM), Met/Val (MV), and Val/Val (VV)) and *PRNP* mutations (mutation usually associated with genetic CJD (gCJD), GSS, and unspecified). The 129 codon genotypes and allele frequencies of gPrDs were determined from the EUROCJD study [25] and the surveillance of prion diseases in Japan [26].

All patients at our center underwent a detailed physical examination and MRI. Fifty-one patients diagnosed with FTD underwent genetic testing and 36 underwent brain ^18^F-fluorodeoxyglucose positron emission tomography/magnetic resonance imaging (^18^F-FDG PET/MRI). For the 136 patients with prion diseases, 133, 70, 14, 32, and 83 patients respectively underwent EEG, CSF 14-3-3 protein test, CSF Tau protein test, ^18^F-FDG PET/MRI, and genetic testing. For clinical and laboratory data, the same demographic and clinical variables were extracted as shown above. Follow-up data were also collected through clinic visits or telephone interviews. The disease duration between symptom onset and death was calculated.

### Laboratory methods

#### Genetic analyses

Genomic DNA was extracted from fresh peripheral blood leukocytes, and whole-exome sequencing (WES) libraries were generated using the Agilent SureSelect Human All Exon V6 Kit (Agilent Technologies, Santa Clara, CA, USA). The detailed procedure has been described in our previous study [27].

#### 14-3-3 protein level test

CSF protein 14-3-3 levels were detected by western blotting at the National Reference Laboratory for Human Prion Diseases, CDC, China, according to the standard operating procedures (SOPs) [28].

#### Tau level test

Total tau protein levels in the CSF samples were measured by enzyme-linked immunosorbent assay (ELISA) (Innotest hTAU-Ag; Fujirebio, Belgium). Tau level higher than 1400 pg/mL was considered positive based on a previous study [29].

#### Electroencephalogram

The CJD subjects received a 2-h EEG using a 21-lead electroencephalographic transducer (Micromed, Italy). The EEG electrodes were placed according to the International 10-20 system. PSWCs were defined according to the criteria published in 1996 [30].

#### Magnetic resonance imaging

All MRI were performed at 3.0 T (Erlangen, Germany) with the following sequences: T1-weighted image (T1WI), T2-weighted image (T2WI), fluid-attenuated inversion recovery (FLAIR), diffusion-weighted imaging (DWI), and diffusion coefficient (ADC) values. Abnormal or normal signal intensity was assessed using DWI and T2 FLAIR in each of the following regions: cortex, basal ganglia, thalamus, and cerebellum.

#### Positron emission tomography

PET scans were performed using a GE Signa PET/MR 3.0 Tesla scanner (GE Healthcare, Milwaukee, WI). ^18^F-FDG-PET images were acquired within 15 min after intravenous injection of ^18^F-FDG (~ 308 MBq) with an uptake time of 30 min. The images were reconstructed using the ordered subset expectation maximization algorithm (OSEMA) with 16 subsets and 4 iterations.

#### Statistical analysis

Statistical analyses were performed using SPSS version 22.0 (IBM, Armonk, NY, USA). Continuous data are represented as the mean ± SD or median (interquartile range) and compared using the *t* test or one-way ANOVA or Mann-Whitney *U* test. Dichotomous data are shown as percentages and were compared using the *χ*^2^ test or fisher test. Two-tailed *P*-value ≤ 0.05 were considered statistically significant when comparing the two groups. A Bonferroni adjustment was used to correct for multiple comparisons with the threshold for significance at 0.025 (two-sided).

## Results

### Demographics characteristics

A total of 49 patients with gPrDs presenting FTD features were identified. The central findings of each patient are summarized in Additional file 2: Table S1. The frequencies of the different features are reported as the relative percentage of cases exhibiting the respective feature (either present or absent) in the primary article. The proportion of males and females was similar (23/49 = 46.9% women, 26/49 = 53.1% men), and the median age of onset and minimum duration of symptoms were respectively 45 years (range 24–78 years) and 5 years (range 0.8–22 years; 18 patients were still alive, and 2 were not reported). Family history was positive in 40/49 (81.6%) patients, 2 had suspected family history of the disease, 6 had no family history, and 1 was unreported (see Table 1).

**Table 1:** Clinical and auxiliary features of gPrDs with FTD phenotype and grouped by codon 129 genotypes

Variables	Total (*N* = 49)	Codon 129 genotypes		*P* values
		MM (*N* = 11)	MV/VV (*N* = 16)	
**Baseline characteristics**				
Female, %	23/49 (46.9)	5/11 (45.5)	11/16 (68.8)	0.226
Age at onset, years, median (IQR)	45.0 (39.5, 53.5)	59.0 (43.0, 66.0)	43.5 (39.3, 60.3)	0.368
Symptoms duration, years, median (range)	5.0 (2.8, 7.0)	4.5 (1.5, 7.0)	5.0 (3.0,6.9)	0.481
Family history, %	40/49 (81.6)	5/11 (45.4)	14/16 (87.5)	0.033
bvFTD, %	37/49 (75.5)	5/11 (45.5)	12/16 (75.0)	0.118
PPA, %	9/49 (18.4)	6/11 (54.5)	2/16 (12.5)	0.225
FTD-ALS	2/49 (4.1)	0	2/16 (12.5)	0.499
**Clinical features of FTD**				
Disinhibition, %	23/40 (57.5)	7/10 (70.0)	4/13 (30.8)	0.062
Apathy, %	27/42 (64.3)	6/10 (60.0)	9/13 (69.2)	0.685
Loss of empathy, %	8/40 (20.0)	4/10 (40.0)	2/13 (15.4)	0.341
Stereotyped/perseverative behavior, %	16/40 (40.0)	4/10 (40.0)	4/13 (30.8)	0.685
Alterations in food preferences, %	10/40 (25.0)	2/10 (20.0)	2/13 (15.4)	1.000
Executive deficits, %	23/40 (57.5)	5/10 (50.0)	10/13 (76.9)	0.378
Speech disorders, %	23/29 (79.3)	9/11 (81.8)	12/15 (80.0)	1.000
**Clinical features of prion diseases**				
Cognitive dysfunction, %	38/41 (92.7)	10/10 (100)	15/15 (100)	1.000
Parkinsonism, %	28/44 (63.7)	7/10 (70.0)	8/15 (53.3)	0.405
Pyramidal signs, %	9/23 (39.1)	4/10 (40.0)	5/11 (45.4)	1.000
Visual signs, %	5/21 (23.8)	1/10 (10.0)	4/10 (40.0)	0.303
Mutism, %	14/39 (35.9)	5/10 (50.0)	5/15 (33.3)	0.405
Seizure, %	8/31 (25.8)	1/10 (10.0)	3/15 (20.0)	0.626
Cerebellar signs, %	7/31 (22.6)	2/10 (20.0)	3/15 (20.0)	1.000
Myoclonus, %	9/40 (22.5)	4/10 (40.0)	2/15 (12.3)	0.175
**Laboratory features**				
PSWCs on EEG, %	0/29 (0)	0/10 (0)	0/8 (0)	1.000
Positive CSF 14-3-3 protein, %	3/10 (30.0)	3/7 (42.9)	0/2 (0)	0.500
Elevated CSF tau protein, %	6/10 (60.0)	4/7 (57.1)	1/2 (50.0)	1.000
Positive RT-QuIC, %	2/3 (66.7)	1/2 (50.0)	1/1 (100.0)	1.000
Frontotemporal atrophy, %	26/29 (89.7)	10/11 (90.9)	10/11 (90.9)	1.000
Hyperintensity on MRI, %	6/24 (25.0)	3/10 (30.0)	1/10 (10.0)	0.582
Frontotemporal hypoperfusion or hypometabolism, %	10/11 (90.9)	4/4 (100.0)	6/6 (100.0)	1.000
Tau-positive pathology	5/6 (83.3)	1/1 (100.0)	2/2 (100.0)	1.000

*CJD*, Creutzfeldt-Jakob disease; *FTD*, frontotemporal dementia; *IQR*, interquartile range; *PRNP*, prion protein gene; *PSWCs*, periodic sharp wave complexes; *RT-QuIC*, real-time quaking-induced conversion assay

### Clinical and auxiliary features of gPrDs with FTD phenotype

The specific clinical and auxiliary features are shown in Table 1. Emotional, personality, or behavioral changes were the most common first symptom with an incidence of 68.9% (31/45), followed by cognitive dysfunction (46.7%, 21/45), speech disorder (13.3%, 6/45), and parkinsonism (6.7%, 3/45). During the course of the disease, the most common symptoms were cognitive dysfunction (92.7%, 38/41), extrapyramidal symptoms (63.7%, 28/44), and pyramidal signs (39.1%, 9/23). No patients exhibited PSWCs on EEG. Typical imaging findings of prion disease, cortical or striatal hyperintensity on DWI, or FLAIR were observed in only 6 patients (25%, 6/24). Radioisotope brain scanning was performed in 11 patients, of which 10 showed cortex hypoperfusion or hypometabolism. Neuropathological findings were reported in 14 cases, spongiform changes were seen in 8 patients, 5 patients presented with multicentric plaques, 2 patients expressed the scrapie form of the prion protein (PrP^Sc^) in the examined tissues, and 1 patient had elevated detergent-insoluble prion protein. Tau-positive pathological changes were observed in 5 patients (83.3%, 5/6).

**Fig. 1:**
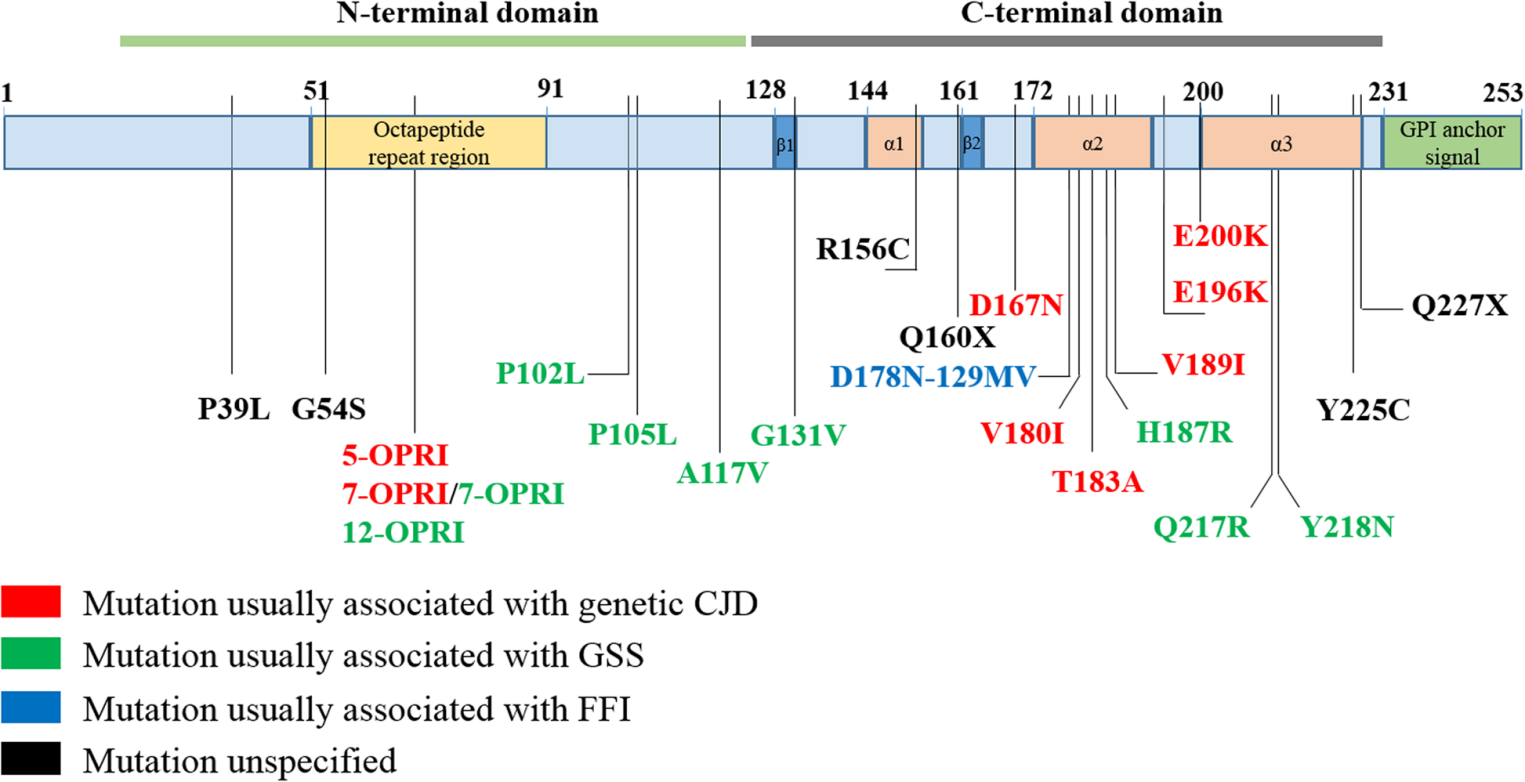
Schematic of *PRNP* mutations associated with FTD phenotypes. Mutations are color-coded based on clinicopathological classification as gCJD, GSS, FFI, or unspecified. OPRI, octapeptide repeat insertion

Twenty point mutations (P39L, G54S, P102L, P105L, A117V, G131V, R156C, Q160X, D167N, D178N-129MV, V180I, T183A, H187R, V189I, E196K, E200K, Q217R, Y218N, Y225C, Q227X) and 3 insertional mutations (five, seven, and twelve octapeptide repeat insertion) of *PRNP* have been reported so far. P39L was the most frequent mutation and was detected in four families with 5 cases, while T183A had the highest number of cases (*n* = 13). Among the 23 mutations, 9 were usually associated with GSS, 8 with gCJD, 1 with FFI, and 6 were unspecified (Fig. 1). Around two-thirds of the mutations are in the C-terminal domain of *PRNP* (codon 125-230).

**Fig. 2:**
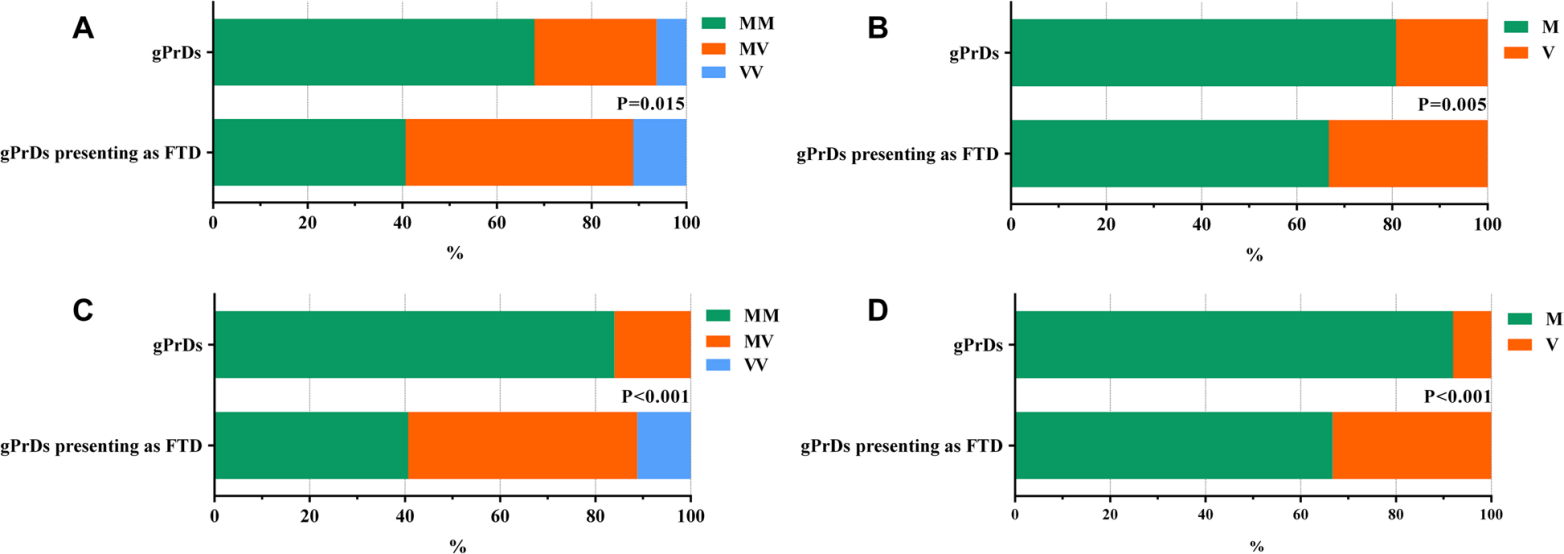
Comparison of the genotypes and allele frequency of codon 129 between gPrDs with FTD phenotype and general gPrDs in Caucasians (**A**, **B**) and East Asians (**C**, **D**). Data of codon 129 for general gPrDs referred from the EUROCJD study [25] and the surveillance of prion diseases in Japan [26]

The patients were also grouped on the basis of codon 129 genotypes (Table 1) and *PRNP* mutations (Additional file 2: Table S1). The frequency of the MM, MV, and VV genotypes at codon 129 were 40.7% (11/27), 48.1% (13/27), and 11.1% (3/27), respectively, and the allele frequency of Met and Val were 66.7% and 33.3%, respectively. The frequency of Val carriers and allele was significantly higher than that reported in the literature for gPrDs in Caucasians (59.3% vs. 32.1%, *P* = 0.015; 33.3% vs. 19.2%, *P* = 0.005) and East Asians (59.3% vs. 16.0%, *P* < 0.001; 33.3% vs. 8.0%, *P* < 0.001) (Fig. 2). Patients with the MV/VV genotype are more likely to have a family history of the disease compared to the MM genotype (87.5% vs. 45.4%, *P* = 0.033).

### Clinical and auxiliary features of gPrDs with FTD phenotype compared to that of FTD/prion diseases

**Fig. 3:**
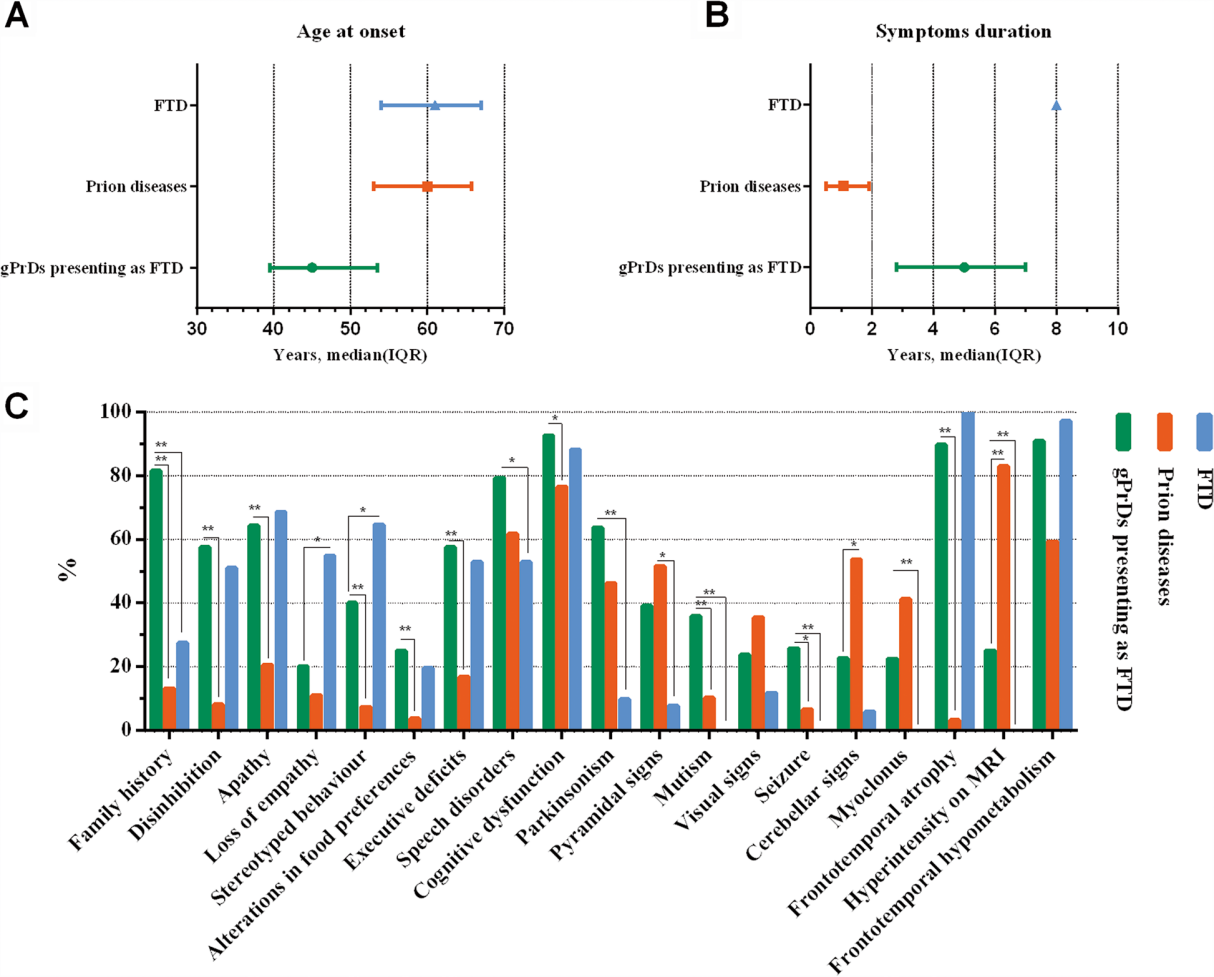
The comparison of age at onset (**A**), symptoms duration (**B**), and main clinical and auxiliary features (**C**) between gPrDs presenting as FTD and FTD/prion diseases. *P < 0.25, **P < 0.001

Of the 51 cases of FTD, 43 were diagnosed as bvFTD, 6 as PPA, and 2 as FTD-ALS. Gene mutations were detected in 23.5% of the total cases and occurred mainly in the *MAPT* gene (17.6%, 9/51), followed by *C9orf72* repeat amplification (2.0%, 1/51), *GRN* gene (2.0%, 1/51), and *FUS* gene (2.0%, 1/51). None of the patients had *PRNP* mutation. The survival of FTD patients were obtained from previous reports since only 2 patients in our study died during the follow-up [31]. Compared to the FTD patients, the gPrD patients with FTD phenotype had an earlier age of onset [median (IQR) 61.0 (54.0, 67.0) vs. 45.0 (39.5, 53.5) years, *P* < 0.001], shorter duration of symptoms [median (IQR) 8.0 vs. 5.0 (2.8, 7.0) years], and a higher incidence of family history (27.5% vs. 81.6%, *P* < 0.001). GPrDs presenting with FTD were also associated with higher rates of parkinsonism (63.7% vs. 9.8%, *P* < 0.001), pyramidal signs (39.1% vs. 7.8%, *P* = 0.001), mutism (35.9% vs. 0%, *P* < 0.001), seizures (25.8% vs. 0%, *P* < 0.001), myoclonus (22.5% vs. 0%, *P* < 0.001), and hyperintensity on MRI (25.0% vs. 0, *P* < 0.001) compared to FTD (Fig. 3, Tables 2, 3, and 4).

**Table 2:** Comparison of baseline features between gPrD patients with FTD phenotype and FTD/prion disease

Variables	GPrDs presenting as FTD (*N* = 49)	FTD (*N* = 51)	Prion diseases (*N* = 136)	*P* values^a^	*P* values^b^
Genetic mutation	49/49 (100.0)	12 (23.5)	17/83 (20.5)	–	–
Female, %	23/49 (46.9)	24 (47.0)	64 (47.1)	0.990	0.988
Age at onset, years, median (IQR)	45.0 (39.5, 53.5)	61.0 (54.0, 67.0)	60.0 (53.0,65.8)	< 0.001	< 0.001
Symptoms duration, years, median (IQR)	5.0 (2.8, 7.0)	–	1.1 (0.5,1.9)	–	< 0.001
Family history, %	40/49 (81.6)	14 (27.5)	18 (13.2)	< 0.001	< 0.001
bvFTD, %	37/49 (75.5)	43 (84.3)	–	0.271	–
PPA, %	9/49 (18.4)	6 (11.7)	–	0.355	–
FTD-ALS, %	2/49 (4.1)	2 (3.9)	–	1.000	–

*bvFTD*, behavioral variant-frontotemporal dementia; *FTD-ALS*, frontotemporal dementia-amyotrophic lateral sclerosis; *PPA*, primary progressive aphasia

^a^GPrDs presenting as FTD vs. FTD

^b^GPrDs presenting as FTD vs. prion diseases

**Table 3:** Comparison of clinical features between gPrD patients with FTD phenotype and FTD/prion disease

Variables	GPrDs presenting as FTD (*N* = 49)	FTD (*N* = 51)	Prion diseases (*N* = 136)	*P* values^a^	*P* values^b^
**Clinical features of FTD**					
Disinhibition, %	23/40 (57.5)	26 (51.0)	11 (8.1)	0.536	< 0.001
Apathy, %	27/42 (64.3)	35 (68.7)	28 (20.6)	0.658	< 0.001
Loss of empathy, %	8/40 (20.0)	28 (54.9)	15 (11.0)	0.001	0.139
Stereotyped/perseverative behavior, %	16/40 (40.0)	33 (64.7)	10 (7.4)	0.019	< 0.001
Alterations in food preferences, %	10/40 (25.0)	10 (19.6)	5 (3.7)	0.538	< 0.001
Executive deficits, %	23/40 (57.5)	27 (52.9)	23 (16.9)	0.664	< 0.001
Speech disorders, %	23/29 (79.3)	27 (52.9)	84 (61.8)	0.019	0.072
**Clinical features of prion diseases**					
Cognitive dysfunction, %	38/41 (92.7)	45 (88.2)	104 (76.5)	0.475	0.022
Parkinsonism, %	28/44 (63.7)	5 (9.8)	63 (46.3)	< 0.001	0.046
Pyramidal signs, %	9/23 (39.1)	4 (7.8)	70 (51.5)	0.001	0.274
Mutism, %	14/39 (35.9)	0	14 (10.3)	< 0.001	< 0.001
Visual signs, %	5/21 (23.8)	6 (11.8)	48 (35.3)	0.197	0.300
Seizure, %	8/31 (25.8)	0	9 (6.6)	< 0.001	0.001
Cerebellar signs, %	7/31 (22.6)	5 (5.9)	73 (53.7)	0.112	0.003
Myoclonus, %	9/40 (22.5)	0	56 (41.2)	< 0.001	0.031

^a^GPrDs presenting as FTD vs. FTD

^b^GPrDs presenting as FTD vs. prion diseases

**Table 4:** Comparison of auxiliary features between gPrD patients with FTD phenotype and FTD/prion disease

Variables	GPrDs presenting as FTD (*N* = 49)	FTD (*N* = 51)	Prion diseases (*N* = 136)	*P* values^a^	*P* values^b^
PSWCs on EEG, %	0/29 (0)	–	40/133 (30.1)	–	0.001
Positive CSF 14-3-3 protein, %	3/10 (30.0)	–	31/70 (44.3)	–	0.393
Elevated CSF tau protein, %	6/10 (60.0)	–	8/14 (57.1)	–	0.899
Positive RT-QuIC, %	2/3 (66.7)	–	–	–	–
Frontotemporal atrophy, %	26/29 (89.7)	51 (100.0)	4 (3.3)	0.044	< 0.001
Hyperintensity on MRI, %	6/24 (25.0)	0 (0)	113 (83.0)	< 0.001	< 0.001
Frontotemporal hypoperfusion or hypometabolism, %	10/11 (90.9)	35/36 (97.2)	19/32 (59.4)	0.417	0.054

*PSWCs*, periodic sharp wave complexes; *RT-QuIC*, real-time quaking-induced conversion assay

^a^GPrDs presenting as FTD vs. FTD

^b^GPrDs presenting as FTD vs. prion diseases

A total of 136 cases of prion diseases, including 119 cases of probable sporadic CJD, 4 of gCJD (*PRNP* T188K), 11 of FFI (D178N/129MM), and 2 of GSS (*PRNP* P102L) were enrolled in this study. GPrDs with FTD phenotype had an earlier age of onset [median (IQR) 45.0 (39.5, 53.5) vs. 60.0 (53.0,65.8) years, *P* < 0.001], longer duration of symptoms [median (IQR), at least 5.0 (2.8, 7.0) vs. 1.1 (0.5, 1.9) years, *P* < 0.001], and a higher incidence of family history (81.6% vs. 13.2, *P* < 0.001) compared to patients with prion diseases. The typical features of bvFTD were less common in patients with prion diseases. Furthermore, GPrDs presenting as FTD had higher rates of cognitive dysfunction (92.7% vs. 76.5, *P* = 0.022), parkinsonism (63.7% vs. 46.3, *P* = 0.046), mutism (35.9% vs. 10.3%, *P* < 0.001), seizures (25.8% vs. 6.6%, *P* = 0.001), and frontotemporal atrophy (89.7% vs. 3.3%, *P* < 0.001) and lower rates of cerebellar signs (22.6% vs. 53.7%, *P* = 0.003), PSWCs on EEG (22.6% vs. 53.7%, *P* = 0.003), and hyperintensity on MRI (25.0% vs. 83.0%, *P* < 0.001) compared to patients with prion diseases (Fig. 3, Tables 2, 3, and 4).

## Discussion

Our study is the first to describe the core clinical and ancillary features of gPrDs with FTD phenotypes, along with their similarities and differences with typical FTD and prion diseases. GPrDs presenting with FTD phenotype are characterized by early-onset and high incidence of inherited cases. In addition, the disease course and clinical manifestations are intermediate of that between FTD and prion diseases, while the auxiliary features were closer to that of FTD. Our findings highlight the apparent phenotypic heterogeneity of gPrDs, which can aid in their early identification.

Despite the overlap with FTD and prion diseases, gPrDs presenting as FTD have certain unique characteristics. First, the overall age of onset is lower, and the majority of patients have a clear or suspected positive family history, which is higher than the 74% reported for genetic FTD and 53% for gPrDs [25, 32]. Second, the disease course and general clinical manifestations are intermediate of FTD and prion diseases. However, the incidence of extrapyramidal symptoms and seizures is higher than that of typical FTD and prion diseases. Extrapyramidal symptoms are not uncommon in FTD either, although they usually appear several years after onset unlike the early presentation in patients with prion disease. In a cross-sectional epidemiological study of FTD in the UK, 64% of the patients with bvFTD presented dyskinesia, 21% with rigidity, and 10% with dystonia, usually at an average of 4.5 years after the onset of symptoms [33]. Third, frontotemporal atrophy and hypoperfusion/hypometabolism are seen in the majority of patients, while typical laboratory features of prion diseases such as PSWCs on EEG and hyperintensity on MRI are relatively uncommon. In our study, no patient showed PSWCs on EEG, and only a quarter of patients showed hyperintensity on MRI. It may be attributed to the inherent low occurrence of PSWCs (less than 10%) in gPrDs and the low incidence of hyperintensity on MRI in gPrDs other than gCJD [25].

A strong genetic component is observed in FTD, wherein 10% of the cases have autosomal dominant inheritance and 30–40% of the cases have a strong family history of dementia [1, 34, 35]. The autosomal dominant mutation in the hexanucleotide repeat expansion in the noncoding region of *C9orf72* and the variants in *GRN* and *MAPT* altogether accounted for 60% of the FTD cases with a genetic basis [36]. In recent years, more autosomal dominant mutations have been identified in FTD, which however are extremely rare and account for less than 5% of global cases [4]. The affected genes include *VCP*, *CHMP2B*, *TARDBP*, *FUS*, *UBQLN2*, *SQSTM1*, *CHCHD10*, *TBK1*, *OPTN*, *CCNF*, and *TIA1* [37]. We identified *PRNP* as another rarely mutated gene in FTD. Therefore, it is recommended to screen for the *PRNP* mutations in the patients with genetically undefined FTD, especially those with early age of onset, positive family history, and presenting typical clinical features of prion diseases and seizures. Overall, *PRNP* mutations may be a rare cause of disorders in the FTD spectrum.

Codon 129 is a known determinant of the phenotype of prion diseases. The homotypic prion protein interactions may occur more rapidly compared to heterotypic interactions, which leads to rapid propagation of prions and/or production of neurotoxic forms of PrP^Sc^, eventually resulting in more severe symptoms and rapid disease progression [38, 39]. In addition, codon 129 also determines the selection of prion strains, which is associated with distinct types of PrPSc and the clinicopathological features [40]. The strain diversity/permissibility in codon 129 Met homozygous patients is greater than that for other genotypes [41]. Patients with sCJD and polymorphic codon 129 genotypes MM, VV, and MV lost 10% of their function in 5.3, 13.2, and 27.8 days, respectively [41]. Therefore, the higher proportion of Val carriers in our study explains the longer disease duration of the FTD phenotype in *PRNP* carriers. Consistent with previous studies, Val carriers usually present with more atypical features [42]. Furthermore, codon 129 polymorphism is strongly associated with disease susceptibility. A few studies have found that heterozygous status may also be associated with susceptibility to PPA [43]. We hypothesized that codon 129 may influence the site of spongiform change, and Val carriers may be more likely to have an initial change in the frontotemporal lobe, followed by frontotemporal atrophy due to the prolonged course of the disease, eventually resulting in the FTD phenotype. Consistent with this hypothesis, spongiform change in the frontotemporal lobe is more obvious in Val homozygous GSS patients [44]. Simone et al. discovered that frontal and temporal lobe lesion severity scores were higher in CJD VV1 patients than in CJD MM1 patients in a semi-quantitative assessment of spongiform change and astrogliosis in 193 CJD brains [45]. However, the pathological features of patients with *PRNP* mutations of different codon 129 genotypes still need to be assessed.

Prion diseases and FTD are neurodegenerative diseases that share common molecular and neuropathological features. In addition, the coexistence of prion disease and FTD in patients suggests an association between the PrP^Sc^ deposits in prion disease and the hyperphosphorylated tau, TDP-43, or FUS aggregates in FTD. Although the presence of tau pathology in *PRNP*-mutated brains is not uncommon, the interaction between PrP and tau protein remains contentious. In animal models, molecular interactions between PrP and tau protein suggest that tau protein may play a role in the biological function of PrP and the pathogenesis of prion diseases [46]. The structural features of PrPSc aggregates lead to the phosphorylation of tau proteins [47], which reflects the frequent coexistence of tau pathology in prion diseases, implying that similar pathogenic mechanisms may exist among diseases encompassing PrPSc deposition to tau aggregation. In recent years, some researchers have advocated classifying both diseases as the same disease spectrum due to the common pathological basis of protein misfolding, aggregation, and spreading [48, 49].

### Limitations

There are several limitations in this study that ought to be considered. First, there were only a few available cases to study, and the description of symptoms was inadequate and pathological findings were missing in many cases, which limited the possibility of drawing definite conclusions. In addition, case reports tend to report exceptional situations and are retrospective, which inevitably introduce some bias. Second, we selected controls from our single center which precluded racial disparities in clinical presentation among diseases. The majority of prion diseases in our study were sporadic CJD rather than the more representative genetic prion disease, which can be attributed to the limited sample size and may have led to some unavoidable bias. Third, some ancillary tests that are necessary for the diagnosis and differential diagnosis of prion diseases are not adequately performed at our center. For example, nearly 40% of the patients did not undergo *PRNP* testing. In addition, the RT-QuIC, which has high sensitivity and specificity in the diagnosis of prion diseases, is still not routinely performed. Fourth, our low autopsy rate, an unfortunate and unavoidable reality in China due to traditional ethical values, may lead to misdiagnosis of prion diseases and FTD and hinder the comparison of prion diseases and FTD-related proteinopathies.

## Conclusions

In summary, gPrDs with FTD phenotype have characteristics intermediate of that of typical FTD and prion diseases. *PRNP* genotyping should be considered for patients with FTD phenotype exhibiting early-onset, family history of the disease and presenting clinical features of prion disease. In addition, the clinical heterogeneity of inherited prion diseases is substantial. Carriers of *PRNP* mutations phenotyped as FTD usually have a longer disease course and higher portion of codon 129 Val genotype, and lack the typical clinical and ancillary features of prion diseases.

## Supplementary Information

**Additional file 1: Figure S1.** Flow chart of the search and selection procedure.

**Additional file 2: Table S1.** The central findings of each patient.

**Additional file 3: Table S2.** Clinical and auxiliary features of gPrDs with FTD grouped by PRNP mutations.

## Data Availability

The dataset generated and analyzed in the current study is available from the corresponding author on reasonable request.

## References

[1] Goldman JS, Farmer JM, Wood EM (2005). Comparison of family histories in FTLD subtypes and related tauopathies. Neurology..

[2] Rohrer JD, Guerreiro R, Vandrovcova J (2009). The heritability and genetics of frontotemporal lobar degeneration. Neurology..

[3] Seelaar H, Kamphorst W, Rosso SM (2008). Distinct genetic forms of frontotemporal dementia. Neurology..

[4] Greaves CV, Rohrer JD (2019). An update on genetic frontotemporal dementia. J Neurol.

[5] Jarmolowicz AI, Chen HY, Panegyres PK (2015). The patterns of inheritance in early-onset dementia: Alzheimer’s disease and frontotemporal dementia. Am J Alzheimers Dis Other Dement.

[6] Jiang Y, Jiao B, Xiao X (2021). Genetics of frontotemporal dementia in China. Amyotroph Lateral Scler Frontotemporal Degener.

[7] Bernardi L, Cupidi C, Frangipane F (2014). Novel N-terminal domain mutation in prion protein detected in 2 patients diagnosed with frontotemporal lobar degeneration syndrome. Neurobiol Aging.

[8] Cupidi C, Bernardi L, Frangipane F (2013). Identification of the novel PRNP gene mutation PRO39LEU in patients affected by frontotemporal dementia. Funct Neurol.

[9] Giaccone G, Indaco A, Moda F (2018). Neuropathological, biochemical, and prion protein aggregation properties of a patient with PRNP P39L variant clinically presenting as frontotemporal dementia. Clin Neuropathol.

[10] Woulfe J, Kertesz A, Frohn I (2005). Gerstmann-Straussler-Scheinker disease with the Q217R mutation mimicking frontotemporal dementia. Acta Neuropathol.

[11] Hall DA, Leehey MA, Filley CM (2005). PRNP H187R mutation associated with neuropsychiatric disorders in childhood and dementia. Neurology..

[12] Clerici F, Elia A, Girotti F (2008). Atypical presentation of Creutzfeldt-Jakob disease: The first Italian case associated with E196K mutation in the PRNP gene. J Neurol Sci.

[13] Kumar N, Boeve BF, Boot BP (2011). Clinical characterization of a kindred with a novel 12-octapeptide repeat insertion in the prion protein gene. Arch Neurol.

[14] Riudavets MA, Sraka MA, Schultz M (2014). Gerstmann-Sträussler-Scheinker syndrome with variable phenotype in a new kindred with PRNP -P102L mutation. Brain Pathol.

[15] Ghetti B, Bonnin J, Garringer H (2018). Neurofibrillary tau pathology and PrP amyloidosis are associated with the PRNP Q160X nonsense mutation. J Neuropathol Exp Neurol.

[16] Del Bo R, Scarlato M, Ghezzi S (2006). Is M129V of PRNP gene associated with Alzheimer’s disease? A case-control study and a meta-analysis. Neurobiol Aging.

[17] He J, Li X, Yang J (2013). The association between the methionine/valine (M/V) polymorphism (rs1799990) in the PRNP gene and the risk of Alzheimer disease: an update by meta-analysis. J Neurol Sci.

[18] Moreno F, Alzualde A, Camblor PM (2011). Prion protein codon 129 polymorphism modifies age at onset of frontotemporal dementia with the C.709-1G>A progranulin mutation. Alzheimer Dis Assoc Disord.

[19] Piguet O, Hornberger M, Mioshi E (2011). Behavioural-variant frontotemporal dementia: diagnosis, clinical staging, and management. Lancet Neurol.

[20] Gorno-Tempini ML, Hillis AE, Weintraub S (2011). Classification of primary progressive aphasia and its variants. Neurology..

[21] Brooks BR, Miller RG, Swash M (2000). El Escorial revisited: revised criteria for the diagnosis of amyotrophic lateral sclerosis. Amyotroph Lateral Scler Other Motor Neuron Disord.

[22] Zerr I, Kallenberg K, Summers DM (2009). Updated clinical diagnostic criteria for sporadic Creutzfeldt-Jakob disease. Brain..

[23] CDC’s Diagnostic Criteria for Creutzfeldt-Jakob Disease (CJD), 2018. Centers for Disease Control and Prevention, National Center for Emerging and Zoonotic Infectious Diseases (NCEZID), Division of High-Consequence Pathogens and Pathology (DHCPP). Available online at: https://www.cdc.gov/prions/cjd/diagnostic-criteria.html.

[24] Wu LY, Zhan SQ, Huang ZY (2018). Expert consensus on clinical diagnostic criteria for fatal familial insomnia. Chin Med J.

[25] Kovács GG, Puopolo M, Ladogana A (2005). Genetic prion disease: the EUROCJD experience. Hum Genet.

[26] Nozaki I, Hamaguchi T, Sanjo N (2010). Prospective 10-year surveillance of human prion diseases in Japan. Brain..

[27] Liu L, Cui B, Chu M (2021). The frequency of genetic mutations associated with behavioral variant frontotemporal dementia in Chinese Han patients. Front Aging Neurosci.

[28] Gao C, Shi Q, Tian C (2011). The epidemiological, clinical, and laboratory features of sporadic Creutzfeldt-Jakob disease patients in China: surveillance data from 2006 to 2010. PLoS One.

[29] Chen C, Hu C, Shi Q (2019). Profiles of 14-3-3 and total tau in CSF samples of Chinese patients of different genetic prion diseases. Front Neurosci.

[30] Steinhoff BJ, Räcker S, Herrendorf G (1996). Accuracy and reliability of periodic sharp wave complexes in Creutzfeldt-Jakob disease. Arch Neurol.

[31] Kansal K, Mareddy M, Sloane KL (2016). Survival in frontotemporal dementia phenotypes: a meta-analysis. Dement Geriatr Cogn Disord.

[32] Ciani M, Benussi L, Bonvicini C (2019). Genome wide association study and next generation sequencing: a glimmer of light toward new possible horizons in frontotemporal dementia research. Front Neurosci.

[33] Coyle-Gilchrist IT, Dick KM, Patterson K (2016). Prevalence, characteristics, and survival of frontotemporal lobar degeneration syndromes. Neurology..

[34] Wood EM, Falcone D, Suh E (2013). Development and validation of pedigree classification criteria for frontotemporal lobar degeneration. JAMA Neurol.

[35] Rosso SM, Donker Kaat L, Baks T (2003). Frontotemporal dementia in The Netherlands: patient characteristics and prevalence estimates from a population-based study. Brain..

[36] Bang J, Spina S, Miller BL (2015). Frontotemporal dementia. Lancet..

[37] Panza F, Lozupone M, Seripa D (2020). Development of disease-modifying drugs for frontotemporal dementia spectrum disorders. Nat Rev Neurol.

[38] Palmer MS, Dryden AJ, Hughes JT (1991). Homozygous prion protein genotype predisposes to sporadic Creutzfeldt-Jakob disease. Nature..

[39] Sandberg MK, Al-Doujaily H, Sharps B (2011). Prion propagation and toxicity in vivo occur in two distinct mechanistic phases. Nature..

[40] Collinge J, Clarke AR (2007). A general model of prion strains and their pathogenicity. Science..

[41] Mead S, Burnell M, Lowe J (2016). Clinical trial simulations based on genetic stratification and the natural history of a functional outcome measure in Creutzfeldt-Jakob disease. JAMA Neurol.

[42] Heinemann U, Krasnianski A, Meissner B (2007). Creutzfeldt-Jakob disease in Germany: a prospective 12-year surveillance. Brain..

[43] Li X, Rowland LP, Mitsumoto H (2005). Prion protein codon 129 genotype prevalence is altered in primary progressive aphasia. Ann Neurol.

[44] Alzualde A, Indakoetxea B, Ferrer I (2010). A novel PRNP Y218N mutation in gerstmann-sträussler-scheinker disease with neurofibrillary degeneration. J Neuropathol Exp Neurol.

[45] Baiardi S, Rossi M, Mammana A (2021). Phenotypic diversity of genetic Creutzfeldt-Jakob disease: a histo-molecular-based classification. Acta Neuropathol.

[46] Han J, Zhang J, Yao H (2006). Study on interaction between microtubule associated protein tau and prion protein. Sci China C Life Sci.

[47] Reiniger L, Lukic A, Linehan J (2011). Tau, prions and Aβ: the triad of neurodegeneration. Acta Neuropathol.

[48] Condello C, DeGrado WF, Prusiner SB (2020). Prion biology: implications for Alzheimer’s disease therapeutics. Lancet Neurol.

[49] Carlson GA, Prusiner SB (2021). How an infection of sheep revealed prion mechanisms in Alzheimer’s disease and other neurodegenerative disorders. Int J Mol Sci..

