# Supplementary figures and images for: Genetic prion diseases presenting as frontotemporal dementia: clinical features and diagnostic challenge
Source: Alzheimers Res Ther. 2022 Jun 29;14:90. doi: 10.1186/s13195-022-01033-4 (PMC9245249; doi:10.1186/s13195-022-01033-4)

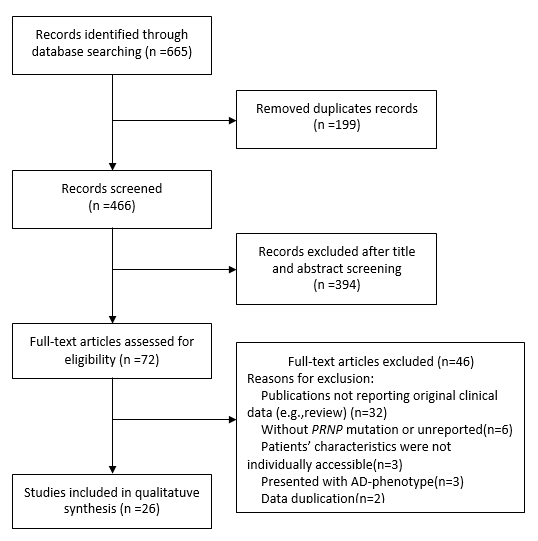

Supplement: Supplementary file 1 — Additional file 1: Figure S1. Flow chart of the search and selection procedure. [file 13195_2022_1033_MOESM1_ESM.tif]
