# Supplementary material for: Genetic prion diseases presenting as frontotemporal dementia: clinical features and diagnostic challenge
Source: Alzheimers Res Ther. 2022 Jun 29;14:90. doi: 10.1186/s13195-022-01033-4 (PMC9245249; doi:10.1186/s13195-022-01033-4)
Supplement: Supplementary file 3 — Additional file 3: Table S2. Clinical and auxiliary features of gPrDs with FTD grouped by PRNP mutations. [file 13195_2022_1033_MOESM3_ESM.docx]

**Supplementary** **Table 2** Clinical and auxiliary features of gPrDs with FTD grouped by *PRNP* mutations

| Variables | *PRNP* mutations | | |
| --- | --- | --- | --- |
|  | gCJD(N=23) | GSS(N=12) | Others(n=14) |
| Baseline characteristics |  |  |  |
| Female, % | 8/23(34.8) | 8/12(66.7) | 7/14(50.0) |
| Age at onset, year, median(range) | 45(24, 78) | 43(36, 61) | 52(30, 75) |
| Symptoms duration, year, median(range) | 4(1.5, 22) | 6.8(0.8, 15.7) | 5(1, 13) |
| Family history, % | 19/23(82.6) | 10/12(83.3) | 11/14(78.6) |
| bv-FTD, % | 20/23(87.0) | 8/12(66.7) | 9/14(64.3) |
| PPA, % | 2/23(8.7) | 2/12(16.7) | 5/14(35.7) |
| FTD-ALS | 0 | 2/12(16.7) | 0 |
| Clinical features of prion diseases |  |  |  |
| Disinhibition, % | 13/20(65.0) | 6/9(66.7) | 4/11(36.5) |
| Apathy, % | 12/21(57.1) | 7/10(70.0) | 8/11(72.7) |
| Loss of empathy, % | 2/20(10.0) | 3/9(33.3) | 3/11(27.3) |
| Stereotyped/perseverative behaviour, % | 10/20(50.0) | 2/9(22.2) | 4/11(36.4) |
| Alterations in food preferences, % | 7/20(35.0) | 0/9(0) | 3/11(27.3) |
| Executive deficits, % | 10/20(50.0) | 7/9(77.7) | 6/11(54.5) |
| Speech disorders, % | 5/6(83.3) | 8/11(72.7) | 10/12(83.3) |
| Clinical features of prion diseases |  |  |  |
| Cognitive dysfunction, % | 15/18(83.3) | 12/12(100) | 11/11(100) |
| Parkinsonism, % | 16/22(72.7) | 6/11(54.5) | 6/11(54.5) |
| Pyramidal signs, % | 3/5(60.0) | 5/11(45.5) | 1/8(12.5) |
| Visual signs, % | 2/5(66.7) | 2/9(22.2) | 1/7(14.3) |
| Mutism, % | 6/16(37.5) | 3/12(25.0) | 5/11(45.5) |
| Seizure, % | 3/9(33.3) | 2/11(18.9) | 3/11(27.3) |
| Cerebellar signs, % | 4/9(44.4) | 3/11(27.3) | 0/11(0) |
| Myoclonus, % | 5/19(26.3) | 2/10(20.0) | 2/11(18.2) |
| Laboratory features |  |  |  |
| PSWCs on EEG, % | 0/14(0) | 0/6(0) | 0/9(0) |
| Positive CSF 14-3-3 protein, % | 2/4(50.0) | 0/2(0) | 1/4(25.0) |
| Elevated CSF tau protein, % | 2/4(50.0) | 2/2(100.0) | 2/4(50.0) |
| Positive RT-QuIC, % | 1/2(50.0) | 0/0 | 1/1(100.0) |
| Frontotemporal atrophy, % | 10/11(90.0) | 7/8(87.5) | 9/10(90.0) |
| Hyperintensity on MRI, % | 3/7(42.9) | 2/8(25.0) | 1/9(11.1) |
| Frontotemporal hypoperfusion or hypometabolism, % | 2/3(66.7) | 4/4(100.0) | 4/4(100.0) |
| Tau-positive pathology | 0/1(0) | 4/4(100.0) | 1/1(100.0) |

CJD, Creutzfeldt-Jakob disease; FTD, frontotemporal dementia; IQR, interquartile range; *PRNP*, prion protein gene; PSWCs, periodic sharp wave complexes; RT-QuIC, Real Time-Quaking Induced Conversion assay

**References** for Supplementary Table 1^1-26^

1 Nitrini R, da Silva LST, Rosemberg S, et al. Prion disease resembling frontotemporal dementia and parkinsonism linked to chromosome 17. *Arquivos De Neuro-Psiquiatria* 2001;59:161-64.

2 Hall DA, Leehey MA, Filley CM, et al. PRNP H187R mutation associated with neuropsychiatric disorders in childhood and dementia. *Neurology* 2005;64:1304-06.

3 Woulfe J, Kertesz A, Frohn I, et al. Gerstmann-Straussler-Scheinker disease with the Q217R mutation mimicking frontotemporal dementia. *Acta Neuropathologica* 2005;110:317-19.

4 Clerici F, Elia A, Girotti F, et al. Atypical presentation of Creutzfeldt-Jakob disease: The first Italian case associated with E196K mutation in the PRNP gene. *Journal of the Neurological Sciences* 2008;275:145-47.

5 Giovagnoli AR, Di Fede G, Aresi A, et al. Atypical frontotemporal dementia as a new clinical phenotype of Gerstmann-Straussler-Scheinker disease with the PrP-P102L mutation. Description of a previously unreported Italian family. *Neurological Sciences* 2008;29:405-10.

6 Alzualde A, Indakoetxea B, Ferrer I, et al. A Novel PRNP Y218N mutation in gerstmann-sträussler-scheinker disease with neurofibrillary degeneration. *Journal of Neuropathology and Experimental Neurology* 2010;69:789-800.

7 Beck J, Poulter M, Hensman D, et al. Large C9orf72 hexanucleotide repeat expansions are seen in multiple neurodegenerative syndromes and are more frequent than expected in the UK population. *American journal of human genetics* 2010;92:345-53.

8 Jansen C, Parchi P, Capellari S, et al. Prion protein amyloidosis with divergent phenotype associated with two novel nonsense mutations in PRNP. *Acta Neuropathologica* 2010;119:189-97.

9 Bernardi L, Anfossi M, Gallo M, et al. Prion protein insertion in a family affected by frontotemporal dementia associated to the PSEN1 V412I mutation. *Clinical Neuropathology* 2010;29:186.

10 Jansen C, Parchi P, Capellari S, et al. A second case of Gerstmann-Sträussler-Scheinker disease linked to the G131V mutation in the prion protein gene in a Dutch patient. *Journal of Neuropathology and Experimental Neurology* 2011;70:698-702.

11 Kumar N, Boeve BF, Boot BP, et al. Clinical characterization of a kindred with a novel 12-octapeptide repeat insertion in the prion protein gene. *Archives of Neurology* 2011;68:1165-70.

12 Cupidi C, Bernardi L, Frangipane F, et al. Identification of the novel PRNP gene mutation PRO39LEU in patients affected by frontotemporal dementia. *Functional Neurology* 2013;28:18.

13 McKnight K, Herron B, Turkington J, et al. Inherited prion disease due to 5-octapeptide repeat insertion. *Journal of the Neurological Sciences* 2013;333:e334-e35.

14 San Millán B, Teijeira S, Rodriguez R, et al. Gerstmann-Strãussler-Scheinker disease. Description of the first case in Galicia. *Clinical Neuropathology* 2013;32:541.

15 Bernardi L, Cupidi C, Frangipane F, et al. Novel N-terminal domain mutation in prion protein detected in 2 patients diagnosed with frontotemporal lobar degeneration syndrome. *Neurobiology of Aging* 2014;35:2657.e7-e11.

16 Mano KK, Matsukawa T, Mitsui J, et al. Atypical parkinsonism caused by Pro105Leu mutation of prion protein: A broad clinical spectrum. *Neurology Genetics* 2016;2:e48.

17 Riudavets MA, Sraka MA, Schultz M, et al. Gerstmann-sträussler-scheinker syndrome with variable phenotype in a new kindred with PRNP -P102L mutation. *Brain Pathology* 2014;24:142-47.

18 Oldoni E, Fumagalli GG, Serpente M, et al. 21) PRNP P39L Variant is a Rare Cause of Frontotemporal Dementia in Italian Population. *Journal of Alzheimer's disease : JAD* 2016;50:353-7.

19 Kenny J, Woollacott I, Koriath C, et al. A novel prion protein variant in a patient with semantic dementia. *Journal of Neurology, Neurosurgery and Psychiatry* 2017;88:891-92.

20 Ghetti B, Bonnin J, Garringer H, et al. Neurofibrillary tau pathology and PrP amyloidosis are associated with the PRNP Q160X nonsense mutation. *Journal of Neuropathology and Experimental Neurology* 2018;77:529-30.

21 Sun Y, Xia M, Yang H, et al. Fatal familial insomnia preliminarily diagnosed as frontotemporal dementia: a case report and literature review. *Chinese Journal of Neurology* 2018;51:294-98.

22 Takayanagi M, Suzuki K, Nakamura T, et al. Genetic Creutzfeldt-Jakob disease with a glutamate-to-lysine substitution at codon 219 (E219K) in the presence of the E200K mutation presenting with rapid progressive dementia following slowly progressive clinical course. *Clinical Neurology* 2018;58:682-87.

23 Bagyinszky E, Yang Y, Giau VV, et al. Novel prion mutation (p.Tyr225Cys) in a Korean patient with atypical Creutzfeldt-Jakob disease. *Clinical Interventions in Aging* 2019;14:1387-97.

24 Di Fede G, Catania M, Atzori C, et al. Clinical and neuropathological phenotype associated with the novel V189I mutation in the prion protein gene. *Acta Neuropathologica Communications* 2019;7:

25 Priemer D, Garringer H, Richardson R, et al. Novel neuropathologic findings in the first american case of frontotemporal dementia associated with the T183A PRNP mutation. *Journal of Neuropathology and Experimental Neurology* 2019;78:569-70.

26 Townley RA, Polsinelli AJ, Fields JA, et al. Longitudinal clinical, neuropsychological, and neuroimaging characterization of a kindred with a 12-octapeptide repeat insertion in PRNP: the next generation. *Neurocase* 2020;26:211-19.
